# Hospital Festivals and Meetings

**Published:** 1895-07-13

**Authors:** 


### HOSPITAL FESTIVALS AND MEETINGS.

### LONDON HOMŒOPATHIC HOSPITAL.

On Tuesday last the new buildings of the London Homœopathic Hospital in Great Ormond Street, just completed, were formally opened by H.R.H. the Duchess of Teck. A great many friends of the hospital were present, and the large ward on the ground floor where the opening ceremony was to take place was filled to overflowing long before the hour fixed. The waiting time (and the Duchess was more than half-an-hour late) was pleasantly filled by listening to the music of the new Blue Hungarian Band, varied by contributions from the band of the City of London Volunteers, who formed the guard of honour outside. Her Royal Highness was received at the entrance by the Earl of Wemyss and March, the Earl of Dysart, Lord Ebury, Lord Newton, Viscount Emlyn, Mr. J. P. Stilwell, Mr. A. E. Chambre, and others, and, after accepting a bouquet from the Hon. Alice Grosvenor, was conducted to the ward. Here an address was read by the chairman of the Building Committee, Mr. Alan E. Chambre, and after a short service, conducted by the Bishop of Stepney and the Rev. Dacre Craven, chaplain to the hospital, and the singing of anthems and a hymn, the architect of the new buildings, Mr. W. A. Pite, presented a key to the Duchess of Teck, who declared the hospital open, and, in so doing, made a pleasant little speech of congratulation. Afterwards Her Royal Highness inspected a portion of the hospital. The visitors were entertained with tea in the "Quin" Ward, and went over the new building. The number of beds in the new hospital is 100. The cost of site, building, and furnishing has amounted to a sum of £45,000, of which £10,000 is still to seek. The cost of maintaining the hospital on its new and improved lines will exceed by some £2,000 that of former days, and it will, therefore, be understood that an increase in subscriptions and donations is much needed.

